# Evaluation of post-discharge engagement for emergency department patients with opioid use history who received telehealth recovery coaching services

**DOI:** 10.1186/s13011-023-00523-4

**Published:** 2023-02-11

**Authors:** Dennis P. Watson, Peter Phalen, Spencer Medcalf, Sarah Messmer, Alan McGuire

**Affiliations:** 1 Chestnut Health Systems, Lighthouse Institute, 221 W Walton St, Chicago, IL 60610 USA; 2 Division of Psychiatric Services Research, University of Maryland School of Medicine, 655 W Baltimore St, Baltimore, MD 21201 USA; 3 Indiana University Health, 950 N Meridian St, Ste 900, Indianapolis, IN 46204 USA; 4 Department of Academic Internal Medicine and Pediatrics, University of Illinois at Chicago, 840 S Wood St, Chicago, IL 60612 USA; 5 Department of Social and Behavioral Sciences, Indiana University Richard M. Fairbanks School of Public Health, 1050 Wishard Blvd, Indianapolis, IN 46202 USA; 6 Richard L. Roudebush VAMC, Health Services Research and Development, 1481 W 10th St (11H) Rm C8108, Indianapolis, IN 46202 USA

**Keywords:** Peer services, Peer recovery coach, Recovery support services, Telehealth, Emergency department, Opioid use

## Abstract

**Background:**

In recent years, emergency departments (EDs) across the nation have implemented peer recovery coach (PRC) services to support patients who use opioids. The majority of such interventions discussed in the literature follow an in-person modality where PRCs engage patients directly at the ED bedside. However, the use of telehealth services in EDs is becoming more popular. These services connect PRCs with ED patients in real-time via secure communications technology, and very little is known about the service- and clinical-based outcomes with which they are associated. The current study sought to assess factors associated with successful post-discharge follow-up of patients with a history of opioid use who received PRC telehealth services while in the ED.

**Method:**

Data come from records for 917 patients who engaged with a telehealth PRC one or more times (1208 total engagements) at 1 of 13 EDs within the same health system. A multilevel Poisson regression model was used to assess the degree to which variables predicted successful post-discharge follow-up, defined as the number of times a PRC successfully spoke with the patient each month after ED discharge.

**Results:**

At least one follow-up was successfully completed by a PRC for 23% of enrolled patients. Significant predictors of successful follow-up included patient employment at baseline (Incidence Rate Ratio [IRR]: 2.8, CI: 2.05–3.9), living in a rural area (IRR: 1.8, CI: 1.04–3.2), PRC provision of referrals (IRR: 1.7, CI: 1.2–2.2), number of ED encounters in the previous 365 days (IRR: 0.99, CI: 0.98–0.99), and duration of the initial PRC telehealth interaction (IRR: 0.87, CI: 0.85–0.88).

**Conclusion:**

Given that relationship development is a key tool in the PRC profession, understanding successful follow-up associated with telehealth engagement has unique importance. The results have potential utility for planning and implementing peer telehealth services in EDs and other locations, which is needed for the development of the PRC profession and the likely expansion of peer telehealth services.

## Background

Drug overdose is a significant and escalating public health problem in the United States, with the Centers for Disease Control and Prevention reporting almost 90,000 people died of an opioid-involved overdose in 2021 [1]. This unprecedented number emphasizes the increasing necessity of interventions to first identify individuals who use opioids and then engage them in recovery support services and related treatment. One promising strategy that has expanded nationally in recent years is embedding peer recovery coaches (PRC) within emergency departments (ED) [2–4]. PRCs are individuals with lived recovery experience who are certified to provide supportive services to people living with a substance use disorder. The current literature on PRC-based ED interventions focuses largely on services delivered directly at the patient bedside [5–9]; however, such services are increasingly being implemented using telehealth modalities [10–13]. More research is necessary to better understand how PRC telehealth interventions may be used to expand ED capacity to effectively serve patients who use opioids.

PRC services are considered evidence-based [3, 14, 15], and prior research has demonstrated PRCs’ ability to improve treatment engagement and retention outcomes [14–16]. These outcomes are often credited to the emotional, instrumental, and informational support provided by PRCs that are beyond the scope of substance use disorder professionals who are strictly clinically focused [17, 18]. There is additional evidence supporting the ability of PRCs who work in EDs to identify patients who use opioids, provide them with harm reduction and supportive services, and link them with appropriate opioid use disorder treatment [6, 9, 19–21]. PRCs can also help bridge a documented gap in the willingness of ED physicians to provide harm reduction interventions or treat opioid use disorder [22, 23]. While there is support for PRC-based interventions to improve treatment engagement and retention [3, 9, 15, 16], it is important to note that standards guiding this developing profession emphasize that services should be collaborative, rather than directive, providing referrals and supports based on the patient’s individualized needs and personal goals [24]. Thus, PRC services should seek to connect patients with a wide range of harm reduction and social supports even (and possibly especially) when treatment linkage is declined. PRCs ability to form long-lasting supportive relationships and connect patients to a broad selection of services and supports is especially valuable in areas with limited treatment options, such as rural locations [25–27].

Patient service engagement is a key challenge for PRC programs that has not been sufficiently explored. Although patients tend to be open to initiating PRC services and report high satisfaction with them [15, 28], rates of sustained engagement are not high. For example, in prior studies, both Welch et al. [29] and Dahlem et al. [30] found PRCs were unable to maintain contact with the majority of patients enrolled in the services they were studying. Since these studies focused on in-person PRC service delivery, they raise even greater concern for the delivery of telehealth PRC services because forming supportive working relationships is a key function of the PRC role [31, 32], and initiating services through a technology-based modality could negatively impact the quality of the initial interaction. While this has not been specifically established with telehealth PRC services, Collins et al. [10] did find that ED providers who moved to telehealth felt it was more difficult to form relationships with patients who used opioids. Additionally, research by Spagnolo et al. [33] identified several barriers to telehealth technology utilization among peer workers, which could futher impact initial and future patient engagement.

The current study seeks to address telehealth PRC services and the impact of the factors that drive PRC follow-up engagement [25]. The study identifed factors associated with the successful post-discharge follow-up of patients with a history of opioid use who engaged in PRC services through a telehealth hub serving multiple EDs across a single hospital system. Research such as this is necessary for pinpointing the most successful factors associated with patient engagement as these results can help inform future effectiveness research [5, 34].

## Methods

This retrospective study is focused on individuals with opioid use history who accepted virtual PRC services when offered to them during an ED encounter. The study was determined not to meet requirements for human subjects research review by the Indiana University Institutional Review Board (2006108993) since the dataset was limited in nature.

### Intervention description

The intervention of focus is part of a larger telehealth program operated by a single Indiana-based hospital system. Program implementation was supported in part by federal opioid response funding distributed through the Indiana Department of Mental Health and Addiction, with these funds being specifically designated for implementing virtual PRC services for ED patients who use opioids. The telehealth program consists of a centrally located telehealth hub with PRCs and other behavioral health professionals available 24 hours a day to participating EDs: all services of focus in this study were PRC-delivered. ED staff activated telehealth services by calling the telehealth hub for patients with an identified need, based either on the presenting problem (e.g., opioid poisoning, intoxication, withdrawal) or information elicited by ED staff during the course of care. When an opioid use issue is identified, staff bring a cart with a video screen to the bedside where they connect the patient with a PRC. Prior to this point, ED staff have provided minimal, if any, information regarding PRC service. At the initial encounter, the PRC describes the program and inquires whether the patient is interested in services. If the patient expresses interest, the PRC proceeds with a conversation aimed at gathering information on the patient’s current substance use, withdrawal symptoms, previous treatment and recovery attempts/pathways, and current needs/desires for linkage to resources. Additionally, the PRC ensures all information for contacting the patient after discharge is included in the electronic health record. After discharge, the PRC refers the patient to their requested treatment or recovery pathways (e.g., outpatient, inpatient, medication-based treatment, 12-step facilitation, detox, etc.). Post-discharge, a PRC attempts follow-up calls at 48 hours; weeks 1 and 2; months 1, 2, 6, and 9; and 1 year. If unable to reach the patient, the PRC will leave a message, if possible. After 3 consecutive unsuccessful attempts, the PRC will stop trying to contact the patient but, if the patient reinitiates contact with the telehealth hub, services will be continued.

In the Indiana-based hospital system studied in this research, 13 EDs implemented 24–7 virtual PRC services as part of a larger telehealth program. Implementation began in September 2018 with services expanding on a rolling schedule through June 2019. Six of the hospitals were located in cities and 7 had rural critical access hospital designations. In all participating EDs, the telehealth PRC service was the only program available for patients presenting with opioid use issues.

### Data and sample

Measures came from two sources. The first source was a database developed to track telehealth hub services, which included PRC services, resources provided, and outcomes related to baseline and follow-up PRC encounters. Details in this database are recorded by the PRC, with information being pulled from the electronic health record or from the discussion with the patient. The second source was the Indiana Network for Patient Care (INPC), which included electronic health record data from hospitals across the state. This allowed us to follow participating patients across a large number of institutions outside the hospital system of focus [35]. These data broadly included information on patients’ ED encounters and hospital admissions, presenting issues, opioid-related diagnoses based on ICD-10 codes, and discharges.

**Fig. 1:**
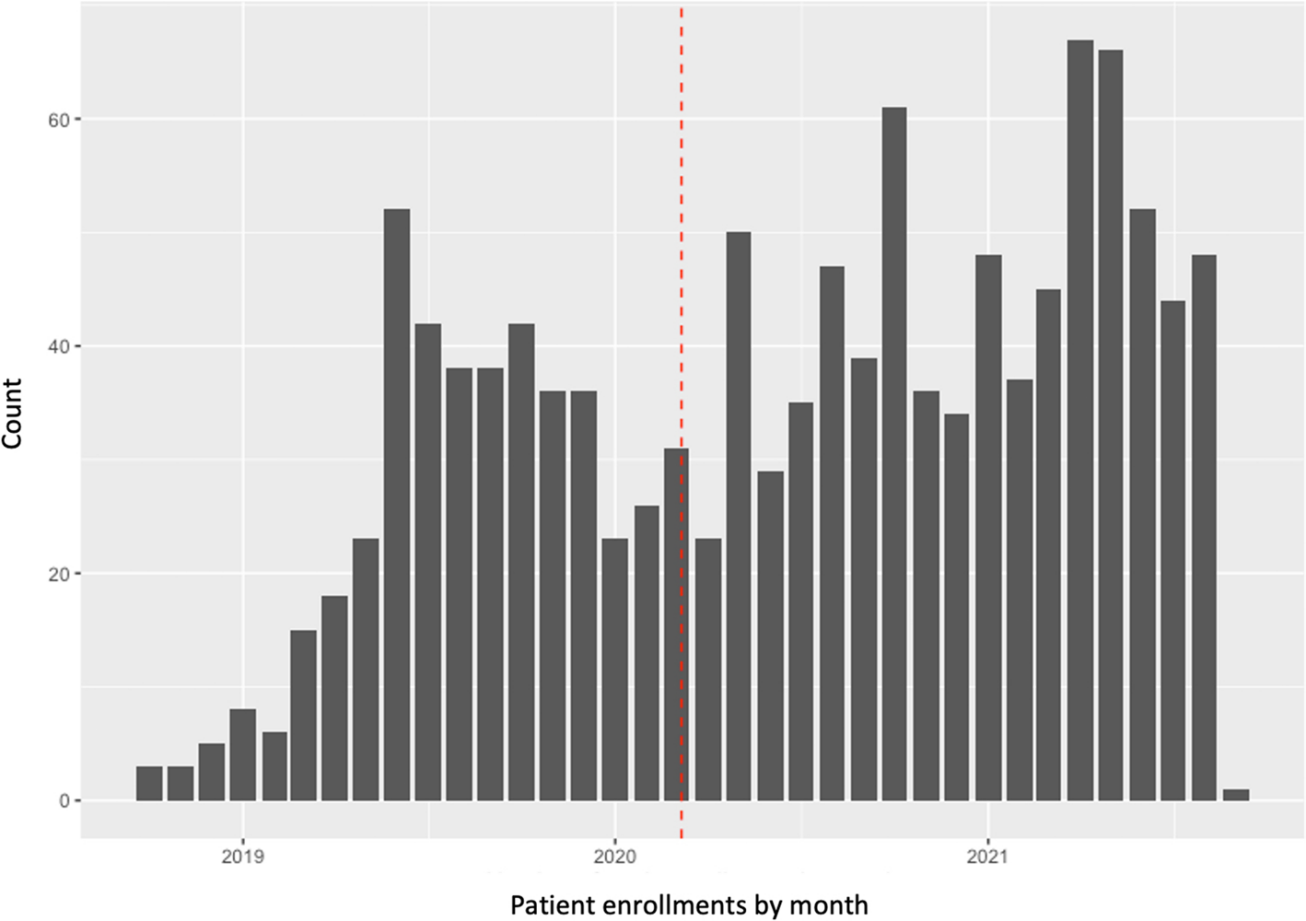
Peer recovery coach emergency department-based patient engagements by month. Notes: Dashed line represents start date of the state’s pandemic emergency order; peer recovery coaches enrolled 423 patients prior to and 792 patients after the emergency order; final month of data collection (September 2021) is not displayed because data collection only persisted through the second day of that month

Our observation window was September 24, 2018 (the date the hub went online at the first ED) through September 2, 2021 (see Table 1 for hospital start dates and total enrollments by hospital and Fig. 1 for number of initial patient engagements by month). To be included in the analysis, a patient must have (a) interacted with a virtual PRC during an ED encounter, (b) accepted enrollment into the PRC program, and (c) had a history of opioid use indicated in one or more locations within the available datasets. The final sample comprised 917 patients who collectively engaged in 1208 baseline (initiating) PRC interactions; some returned to an ED during the follow-up period and re-enrolled in PRC services during the subsequent encounter.

**Table 1:** Setting, month of first peer telehealth enrollment, and total number of patients by site

Hospital	Rural^a^	Month	Total number of patients initiating baseline peer recovery coach interactions
Site 1	Yes	October 2018	68
Site 2	Yes	November 2018	67
Site 3	Yes	January 2019	78
Site 4	No	January 2019	17
Site 5	No	March 2019	126
Site 6	No	March 2019	204
Site 7	No	April 2019	148
Site 8	Yes	April 2019	37
Site 9	Yes	May 2019	18
Site 10	No	May 2019	367
Site 11	Yes	May 2019	30
Site 12	No	June 2019	28
Site 13	Yes	June 2019	20

^a^Rural classification is based on hospital designation as a critical access hospital. All other hospitals were located in cities

### Variables

The primary outcome—the rate of successful follow-ups recorded in the PRC tracking database—is defined as the number of times a PRC successfully spoke with the patient each month of attempted follow-up. Attempts to reach patients for follow-up were recorded in the telehealth hub’s database, with information indicating whether or not they were successful. Within the available datasets, this served as the best factor for assessing the PRC program’s success at facilitating collaborative and engaging relationships with patients, a key function of their professional role [33].

Demographic predictors pulled from the INPC database included: patient age, sex (male versus female), race (White vs. non-White; Black vs. non-Black), and ethnicity (Hispanic versus non-Hispanic). Additional demographic information came from the PRC database and included: employment at baseline (yes vs.no), insurance status (self-pay vs.insured), and whether the patient lived in a metro, small, or rural area as defined by applying rural-urban commuting area codes to patients’ home zip codes (United States Department of Agriculture, 2020). Also examined were predictors related to the current ED encounter recorded in the PRC database which included: naloxone administration prior to ED encounter as an indication of an opioid poisoning (yes vs. no); opioids as the primary reason for the ED encounter (yes vs. no); PRC-provision of referrals (yes vs. no) and the specific type of referrals provided; and the duration of the initial virtual PRC interaction. The final two predictors included patients’ self-reported route of drug administration recorded in the PRC database (four binary variables indicating intravenous, oral ingestion, smoking, inhalation/snorting), and the number of ED encounters recorded in the year prior to telehealth PRC enrollment (as drawn from the INPC data).

### Analysis

First, we examined the sample composition and pattern of follow-up encounters with PRCs with descriptive statistics and exploratory plotting. As conditional mean and variance and were approximately equal, we used a Poisson regression model to assess the degree to which variables of interest predicted the rate of successful follow-up [36]. The regression was multilevel, with rounds of recovery coaching embedded within patients to account for those patients who enrolled in recovery coaching multiple times. We analyzed follow-up attempts as multilevel by embedding them within patient because patient ID accounted for significant variance in rates of successful follow-up. We did not embed patients within hospital sites because site effects were demonstrated to be non-significant. We also did not embed observations within PRCs because patients in this program are not assigned to specific PRCs. Relatedly, preliminary analyses identified a significant effect of a single PRC (out of twelve) whose outreach attempts were associated with substantially lower success rates; however, this PRC was the only coach who consistently worked overnight shifts. Therefore, we decided to interpret the effect as representing a decreased likelihood of successful follow-up for PRC coaching initiated between the hours of 10 pm and 8 am, rather than the effect of the identity of the PRC.

We adjusted the model to account for the large degree of variation in duration of the follow-up period among patients. The duration of the attempted follow-up period depended on on several factors: (1) patient rehospitalization resulting in a new round of PRC services and ending the previous services and follow-up period; (2) patients reaching the end of the 1 year telehealth service program; (3) patient death; and (4) the study observation window ending during the patient’s follow-up period. Adjusting for these factors was important for distinguishing between time periods in which patients were not contacted due to eligibility versus time periods when patients were eligible but not contacted. To achieve this, we included an offset to represent the number of months (or fractions of months) when follow-up was possible. With this adjustment, the Poisson regression can be interpreted as predicting the number of successful contacts per month of attempted outreach. We did consider a zero-inflation model to account for the large proportion of participants without any successful follow-ups. This option was ultimately rejected because (a) the data were approximately Poisson distributed despite the high proportion of zeros and (b) the binomial component of zero-inflation models does not allow for the inclusion of the offsets described above [37], which are important for modeling the specific underlying study design.

Additional analyses was performed to determine whether there were overall changes in rates of successful follow-up due to the COVID-19 pandemic, controlling for changes over time. Corresponding with Indiana’s initial state of emergency order, March 6, 2020 was used as the start date for the pandemic. We also added another interaction term and re-ran the Poisson regressions (described above) to assess whether the impact of variables of interest changed following the pandemic’s onset.

All data processing, modeling, and graphing was performed using the R version 4.1.3 [38], with multilevel regression analyses performed using the *lme4* R package version 1.1.27.1 [39].

## Results

### Sample characteristics

Table 2 displays characteristics for the 917 patients whose 1208 baseline telehealth encounters comprised the sample. As demonstrated, the majority were male, white, and non-Hispanic. Table 3 displays characteristics of these patients at the time of the baseline telehealth encounter: patients were more likely to be unemployed, have public insurance, live in a metro area, and received at least one referral from the PRC. Intravenous and oral drug use were the most preferred routes of administration. The average number of ED encounters in the previous 365 days was 10.8 (range 0–156) and the average baseline call time with the PRC was 22.5 minutes (range 1–274).

**Table 2:** Patient characteristics (*N* = 917)

Variable	Mean	Standard Deviation
Age (at first encounter)	35.2	10.9
	***n***	**Percent (%)**
Sex		
Female	341	37
Male	576	63
Race		
White	865	94
Black	42	4.5
Other	5	0.5
Unknown	5	0.5
Ethnicity		
Non-Hispanic	890	97
Hispanic	23	2.5
Unknown	4	0.5

**Table 3:** Patient characteristics at baseline telehealth encounter (*N* = 1208 encounters)

Variable	***n***	%
Employed		
Yes	285	24
No	807	67
Unknown	116	9
Insurance status		
Public Insurance	1061	87.8
Uninsured	141	11.7
Private Insurance	3	0.2
Unknown	3	0.2
Area living in^a^		
Metro	864	72
Small	165	14
Rural	78	6.5
Opioid-related ED encounter		
Yes	203	16.9
No	1005	83.2
Naloxone administered		
Yes	200	16.6
No	998	82.6
Unknown	10	0.8
Route of administration		
Intravenous	466	38.6
Oral ingestion	432	35.8
Inhalation/Snorting	188	15.6
Smoking	111	9.2
Unknown	11	0.9
Peer coach provided referral		
Yes	902	74.7
No	306	25.3
	**Mean**	**Standard Deviation**
Number ED encounters in previous 365 days	10.8	17.9
Baseline telehealth interaction duration (in minutes)	22.5	21.4

^a^These categories are not comprehensive and therefore add up to less than 100%

### Follow-up contacts

Two hundred and seventy-nine baseline encounters (24%) resulted in at least one successful follow-up PRC contact. Among these, the last successful follow-up contact occurred a median of 14 days after the baseline encounter (mean = 43 days, range of < 1 day to 420 days).

### Predictors of successful follow-up

Table 4 displays the regression analysis results. Significant predictors in the model included employment status, ruralness, PRC provision of referrals, number of ED encounters in the prior year, and duration of the initial PRC telehealth interaction. Those employed at baseline had a 190% greater rate of successful follow-ups. Living in a strictly rural area (i.e., a USDA code of “rural” as opposed to simply “small”) was associated with an 80% greater rate of successful follow-up. Receiving at least one service or treatment referral from the PRC during the initiating interaction resulted in a 70% greater rate ratio of follow-up success, although the number of referrals made by the PRC was not statistically significant. Drilling further down into referrals, outpatient treatment was the only referral type with its own significant effect, approximately doubling the rate of successful follow-up (Incidence Rate Ratio = 2.1, CI: 1.6–2.7). The average PRC baseline call duration was 23 minutes and, for each additional minute of interaction beyond the average, the rate of successful follow-up *decreased* by 13%.

**Table 4:** Rate ratio of successful follow-ups per month

Predictor	Rate Ratio	95% CI
Age	0.99	0.98–1.01
Sex (Male)	0.91	0.68–1.23
Race		
White (versus non-White)	0.97	0.52–1.82
Black (versus non-Black)	0.87	0.42–1.79
Employed	2.84	2.05–3.95*
Insured (versus uninsured)^a^	1.14	0.76–1.71
Living location		
Metro	0.89	0.64–1.22
Small	1.47	0.98–2.19
Rural	1.83	1.04–3.21*
Opioid-related baseline ED encounter	1.22	0.86–1.73
Narcan administered pre-baseline encounter	1.18	0.84–1.68
Preferred route of administration		
Intravenous	0.87	0.66–1.16
Oral ingestion	1.15	0.85–1.54
Inhalation/Snorting	1.27	0.89–1.82
Smoking	0.64	0.38–1.07
Total referrals (number)	1.08	0.99–1.18
Any referrals (at least one versus none)		
All referral types	1.64	1.20–2.24*
Inpatient services	0.77	0.51–1.14
Residential services	0.62	0.32–1.23
Outpatient treatment	2.08	1.60–2.71*
Detox	0.75	0.49–1.15
Peer support group	1.38	0.96–2.00
Medication for opioid use disorder	1.39	0.78–2.49
Number ED encounters in previous 365 days	0.99	0.98–0.99*
Baseline telehealth interaction call (in minutes)	0.87	0.85–0.88*

^a^ > 99% of insurance was public insurance; *p < 0.05

### Impact of COVID-19

**Fig. 2:**
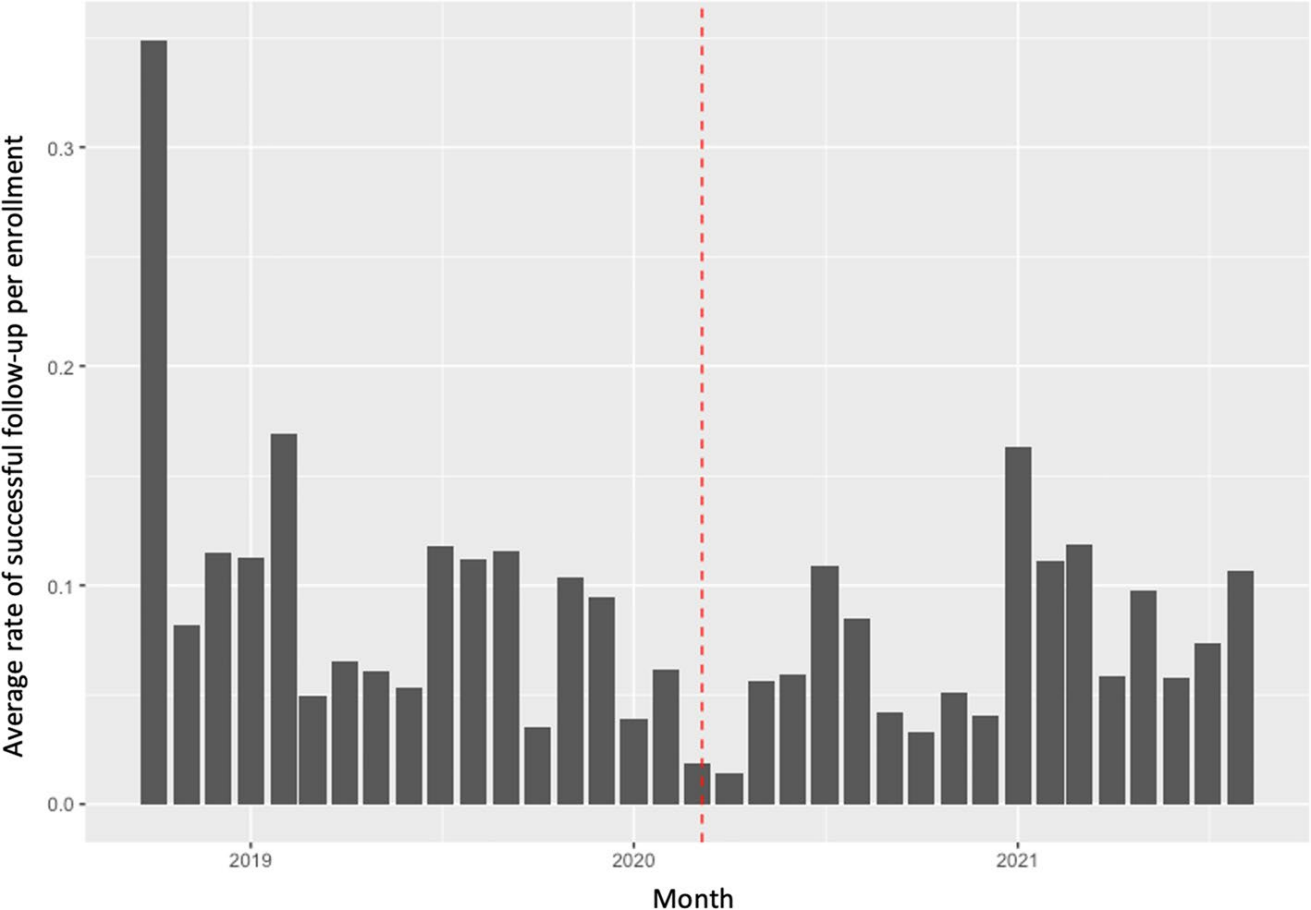
Average rate of successful follow-up for all enrollments initiated by month. Note: Dashed line represents start date of the state’s pandemic emergency order

Figure 2 displays the average rate of successful follow-up for all enrollments initiated within a given month. While not easily observed in the figure, modeling suggests the rate of follow-up was 45% lower (95%CI: 6–68%) on average for enrollments occurring in the period after Indiana’s pandemic emergency order. We also tested for interactions between predictor variables of interest and the time before versus after the pandemic to explore whether the pandemic impacted the strength of association between the predictor variables and follow-up rates. Only baseline call-time time duration had a significant interaction with the pandemic (Incidence Rate Ratio = 0.87, CI: 0.83–0.90), with greater call-time being more strongly associated with decreased follow-up rate for people recruited after the emergency order.

## Discussion

The present study examined patterns of successful follow-up among ED patients with a history of opioid use who enrolled in a telehealth PRC intervention. Twenty-three percent of patients had at least one successful PRC follow-up call. Statistically significant predictors of greater rates of successful outreach during the follow-up period include patient employment at baseline, living in a rural area, and PRC provision of referrals. Number of ED encounters in the previous 365 days and longer duration of the initial PRC telehealth interaction were associated with decreased rate of successful follow-up. While rates of successful follow-up tended to be lower after the COVID-19 pandemic’s onset, only baseline call duration was determined to be significantly impacted, with longer call times associated with less follow-up success. Further research is necessary to better understand the exact manner in which these factors might impact PRC telehealth effectiveness, but some potential interpretations are suggested below.

The finding that patients employed at baseline had higher follow-up rates is consistent with research demonstrating the association between employment and better treatment and recovery outcomes [40–42]. In the case of the current study, it is also possible that employed patients had more consistent and reliable acccess to a working telephone, as phone access is often a barrier to telehealth and treatment engagement for this population [10, 43, 44].

A review of the communications with program staff point toward two possible reasons for the observed association between patients who received referrals at the baseline telehealth interaction and follow-up success (personal communication, April 1, 2022). First, PRCs only provided referrals to patients who indicated interest, suggesting referred patients had slightly higher motivation toward recovery, which has been shown to be predictive of service engagement and outcomes for opioid and other substance use disorders [45–47]. Specifically regarding intensive outpatient treatment programs, PRCs referred patients to programs within the same hospital system when possible, thereby accommodating follow-up with these patients.

Given the greater availability of resources for patients in urban areas, it was not expected to find higher follow-up success rates for those living in rural areas. While it is not possible to state exactly what might be driving this association, it may be rural patients have some pre-existing familiarity and comfort with telehealth given such services are historically more utilized in these areas [48]. Research has also shown that patients in rural areas have more difficulty accessing opioid use disorder treatment and supports, which is often related to the lack of available methadone and buprenorphine providers in their areas [25–27]. Therefore, it is also possible rural patients may have continued to rely on the telehealth PRC program as a form of recovery support due to lack of other options in their communities. Indeed, a prior Indiana-based study of several PRC ED interventions for patients who used opioids identified the lack of local treatment options as a considerable implementation barrier for those programs in rural hospitals [27].

While not empirically proven, prior literature has suggested that the post-overdose period is a particulary salient point during which survivors might be more receptive to treatment and recovery supports [34, 49, 50]. However, naloxone administration—which would be indicative of an overdose—was not significantly associated with increased follow-up success in the current study, although the raw effect was in this direction. It is possible that an opioid-related health crisis is a motivating enough event without having to be overdose-specific. Future work in this area could seek to identify associations between presenting problems and treatment motivation, thus better informing PRC and other professionals’ patient engagement strategies. This finding, coupled with the fact that the majority of opioid-related ED encounters are not overdose-related, indicate a need for ED interventions to extend their reach beyond overdose survivors [12].

The observed relationship between follow-up success and both prior year ED utilization and greater duration of the baseline telehealth interaction could be due to greater case complexities of these patients. Prior research has demonstrated high-utilizing ED patients tend to have longer substance use histories, more high-risk substance use behaviors and associated health consequences, and more complex health issues [51–53]. The telehealth program staff involved in the present study indicated patients with more complex problems tended to have longer baseline interactions because more attention was required to address treatment barriers (personal communication, Feburary 18, 2022). Likewise, the drop in follow-up success following the state’s pandemic order could have also been the result of increased complexities resulting from disruptions the publilc health emergency was having on peoples' lives [54, 55], and this might also account for the significant interaction observed between call time and the pandemic emergency order. The overall rate of successful follow-up observed was less than that seen in two prior studies of peer-based ED interventions conducted by Dahlem et al. [30] and Welch et al. [29] that saw intial follow-up rates of 49 and 33% respectively. These interventions differed from the current one in that they delivered in-person peer services rather than via telehealth. Also and as previously discussed [10], it is possible telehealth services pose some additional difficulty to forming trusting therapeutic relationships with patients who use opioids. One potential way to improve follow-up despite this relationship barrier would be to combine telehealth PRC services with immediate buprenorphine induction within the ED [56, 57], a practice that was not standard in any of the EDs included in this study. That said, success of such an approach depends on the availability of providers who are willing or able to treat patients with opioid use disorder, a potential concern for rural hospitals.

## Limitations

The primary limitation of this study is the use of retrospective health services data. Information was not always consistently recorded in the electronic health record or telehealth database. This also restricted our investigation to information captured in the available service records, which limited our ability to investigate potential confounding factors. Additionally, the records did not provide any information on the recency of substance use behaviors. While this could have been overcome by limiting the analysis to patients with drug overdose/poisoning events, it would likely have overlooked the majority of people who use opioids and receive ED care [12].

Using a service-based outcome (i.e., follow-up rates) does not provide information regarding ultimate clinical effectiveness of the intervention; however, this is a common limitation of health services research studies when detailed clinical outcome data are not recorded within available health records. Furthermore, retention is a prerequisite for clinical effectiveness of long term recovery support interventions, and identifying factors that support or hinder retention opens the door for potential quality improvement strategies. Relatedly, while linkage to medications for addiction recovery (e.g., methadone, buprenorphine, naltrexone) has been an outcome of focus in studies of ED-based opioid interventions, this would not have been an appropriate outcome of focus for this study considering: (a) as appropriate for the profession [33], telehealth PRCs’ role in this program was to assist patients to follow their chosen recovery paths and only a small proportion (6%) chose medication-based treatment options; (b) it is valuable (and possibly more so) to link clients to harm reduction and recovery supports when medication-based treatments are not available or desired; and (c) the rural areas—in which more than half the hospital sites were located—lacked robust options for medication-based treatment for opioid use disorder. Regarding this last point, the average distance to the nearest city with a licensed opioid treatment program/methadone clinic for rural sites was 30 miles (range = 22–51 miles) with an average driving time of 41 minutes in one direction (range = 24–73 minutes), and the average number of providers waivered to prescribe buprenorphine in their immediate areas was 1.7 (range = 0–9), with 4 sites having no waivered providers in their areas [58].

A final weakness is the sample composition being overwhelmingly white and non-Hispanic, which is representative of the state of Indiana within which the study was conducted but less representative of the country as a whole. Strengths of the study include having longitudinal data from a robust health information exchange, allowing us to track ED encounters across a number of hospital systems within the state. While effects of the intervention might have varied across the EDs, the provision of services from the same telehealth hub helped assure there was reasonable consistency of intervention delivery across hospitals.

## Conclusion

This study identified factors associated with successful follow-up of ED patients with an opioid use history who engaged with a telehealth PRC. Such information could be useful for helping to develop competencies and skills of telehealth PRCs, which is a recognized need [29]. For instance, being aware of potential barriers to follow-up could result in different approaches to engagement or the collection of more robust information that could be used to locate the patient at a later date. This information will be useful considering telehealth’s ability to provide needed services to rural areas and the need and desire for ED telehealth services is likely to continue despite the pandemic’s waning.

## Data Availability

The data that support the findings of this study are available from Regenstreif Institute but restrictions apply to the availability of these data, which were used under license for the current study and are not publicly available. However, data are available from the authors upon reasonable request and with permission of Regenstreif Institute.

## Abbreviations

ED: Emergency department
PRC: Peer Recovery Coach
